# Bridging Machine Learning and Event‐Based Analyses to Assess Maternal Contingent Responses to Infant Nondistress and Distress Vocalizations From Home Audio Recordings

**DOI:** 10.1111/desc.70251

**Published:** 2026-08-12

**Authors:** Kexin Hu, Xulin Fan, Yannan Hu, Jialu Li, Bethany Lee, Mark Hasegawa‐Johnson, Nancy L. McElwain

**Affiliations:** ^1^ Department of Human Development and Family Studies University of Illinois Urbana‐Champaign Urbana Illinois USA; ^2^ Department of Electrical and Computer Engineering University of Illinois Urbana‐Champaign Urbana Illinois USA; ^3^ Department of Psychological and Brain Sciences Indiana University Bloomington Bloomington Indiana USA; ^4^ College of Information Science University of Arizona Tucson Arizona USA; ^5^ Beckman Institute for Advanced Science and Technology University of Illinois Urbana‐Champaign Urbana Illinois USA

**Keywords:** home audio recordings, infant vocalizations, maternal contingent responses, machine learning algorithms

## Abstract

Caregiver‐infant vocal interactions are foundational for early development, yet most evidence is derived from brief laboratory or home‐visit observations and assessing caregiver–infant vocal contingency in everyday settings requires scalable methods. To this end, we integrated machine learning (ML) with traditional event‐based analyses to measure maternal contingent vocal responsiveness assessed from daylong audio recordings in the home. In Study 1 (*N* = 61 infants; 1–18 months, *M_age_* = 9.43 months), we evaluated a wav2vec2‐based audio tagging algorithm to detect infant nondistress and distress vocalizations and caregiver vocalizations. Performance was high when tested against human annotations. In Study 2 (*N* = 56 infants; 1–10 months; *M_age_* = 6.61 months), we applied the algorithm to daylong home audio recordings. Maternal response rates based on ML‐generated labels showed strong correspondence with those based on human‐annotated labels, although a consistent positive bias was observed (ML > human). Response‐rate distributions tended toward higher values in the 20 most voluble segments relative to all available mother–infant interaction segments. Additionally, on average, the 20 most voluble segments (vs all available segments) produced higher maternal response rates but lower base‐rate‐adjusted estimates of contingency. Response rates increased with lag window, and in most cases, the sharpest increase was observed between 1 and 2 s, with diminishing returns thereafter. Together, these findings support ML‐derived vocalization labels for scalable contingency estimation and underscore analytic decisions regarding sampling and lag window when estimating maternal contingent responsiveness from daylong home recordings.

## Summary

- Integrated developmental theory and computational modeling to develop and validate an ML algorithm labeling infant and maternal vocalizations from daylong home recordings with high accuracy.
- Demonstrated that ML‐derived labels preserve rank‐order individual differences in maternal contingent responsiveness despite a systematic bias in absolute levels.
- Maternal contingency estimates varied by behavioral sampling strategy, with highly voluble segments yielding higher response rates but lower base‐rate‐adjusted estimates than all available segments.
- Lag windows also shaped contingency estimates, with shorter windows concentrating maternal responses and longer windows capturing additional responses but with diminishing returns.

## 1 Introduction

Infant development is shaped by dynamic interactions with caregivers, and microanalytic methods used to probe the dynamic structure and timing of mother–infant interactions have a rich and long history in developmental science (see Lourenço et al. 2021; Provenzi et al. 2018). This body of work has yielded numerous and important insights into real‐time processes underlying social‐emotional (e.g., Beebe et al. 2010), cognitive (e.g., Jaffe et al. 2001), and language (e.g., Luchkina and Xu 2024) development, yet ecological validity is hampered by brief assessments, typically conducted in the lab, and/or small samples. Advances in wearable technology and machine learning (ML) approaches allow researchers to overcome these hurdles and capture microtemporal processes of mother–infant interactions in real‐world settings and across longer periods of time than has been previously possible (see de Barbaro 2019; Lourenço et al. 2021; Messinger et al. 2022). Given the primacy of vocalizations as the means by which infants elicit maternal caregiving (Bornstein et al. 2015; Hsu and Fogel 2003) and mothers respond to infant cues (van Egeren et al. 2001; Zhang et al. 2024), advances in daylong audio recording technologies (e.g., Language Environment Analysis [LENA] system; Gilkerson et al. 2017; Xu et al. 2009), in particular, have transformed the traditional study of parent‐infant vocal interactions by enabling large‐scale, ecologically valid analyses of naturalistic home environments (e.g., Bergelson et al. 2023).

In this report, we aim to bridge advances in wearable technology and data‐driven ML approaches with traditional event‐based analyses for studying mother–infant interaction. In doing so, we present findings from two interrelated studies. In Study 1, we present a novel ML algorithm that labels infant nondistress and distress vocalizations as well as maternal vocalizations from daylong audio recordings, and we evaluate its performance against gold‐standard human annotations. In Study 2, using the validated ML‐generated labels as a foundation, we first evaluate whether maternal contingent responsiveness computed from ML labels closely corresponds to that computed from human annotations. We then examine how estimated maternal responsiveness derived from ML labels varies as a function of different strategies for sampling behavior (i.e., 20 most voluble segments vs. all available segments) and different lag windows (i.e., 1–5 s) used to define a contingent response.

## 2 Study 1

Speaker diarization and vocalization classification are key signal‐processing tasks that can be leveraged for a more comprehensive and fine‐grained understanding of mother–infant vocal interactions. Speaker diarization aims to identify who spoke when, whereas vocalization classification distinguishes different types of vocalizations for a given speaker. Early speech processing methods have relied primarily on statistical models due to limited labeled datasets and computation resources. With the rise of deep learning (DL), researchers have started building DL‐based models via supervised learning using labeled data. For speaker diarization, end‐to‐end neural diarization (EEND) models formulated this task as a multi‐label multi‐classification problem (Fujita et al. 2019). EEND introduces a single DL‐based model and directly optimizes diarization accuracy by predicting speaker activity given each audio frame, assigning 1 for an active speaker and 0 for an inactive speaker (see Lavechin et al. 2020, for an example of diarization of family home recordings). For vocalization classification, utterance‐level global features, either extracted from entire utterances or aggregated from frame‐level local features using global pooling, play a critical role in capturing overall vocalization patterns. Frame‐level features may also contribute as they capture temporal variations and paralinguistic cues. Interspeech Computational Paralinguistic (COMPARE) Challenges often feature multiple vocalization classification tasks relevant to adult‐infant interactions (e.g., infant cry or sound classification, adult addressee classification, Schuller et al. 2017, 2018, Schuller et al. 2019), and baseline models usually extract a feature set consisting of both utterance‐ and frame‐level features for each vocalization. These feature sets are used to train traditional ML models (e.g., Support Vector Machines, K‐Nearest Neighbors) or neural network‐based models (e.g., feedforward networks, convolutional neural networks) to predict vocalization labels.

Supervised learning models require abundant labeled data, but annotating naturalistic family audio is a labor‐ and time‐intensive task. One representative supervised approach for annotating naturalistic family audio is the Voice Type Classifier (VTC; Lavechin et al. 2020). Trained on a large, diverse, child‐centered annotated corpus (approximately 260 h) spanning 10 languages, VTC segments naturalistic audio into five speaker categories: key child, adult male, adult female, other child, and unknown source. In evaluations, VTC substantially outperformed LENA. However, approaches such as VTC remain fundamentally constrained by the availability of extensive human annotation. To address the challenge of labeled data sparsity, speech foundation models (SFMs) have been developed to enhance downstream speech processing tasks given a limited amount of labeled data via (1) *self‐supervised learning* by pretraining on thousands of hours of unlabeled audio data, such as wav2vec 2.0 (W2V2; Baevski et al. 2020), HuBERT (Hsu et al. 2021), and WavLM (Chen et al. 2022), or (2) *weakly supervised learning* by pretraining on millions of hours of weakly labeled audio with imperfect labels, such as Whisper (Radford et al. 2023). SFMs are well‐suited for developing under‐resourced child‐centered speech processing applications. In this report, we use W2V2, which was originally developed for adult speech processing tasks by first pretraining on large‐scale amount of adult speech. Once pretrained, W2V2 can be fine‐tuned on smaller labeled datasets for applications such as automatic speech recognition (Baevski et al. 2020), speech emotion recognition (Pepino et al. 2021), and speaker diarization (Mishra et al. 2023).

Although W2V2 has demonstrated strong performance in adult speech processing, its effectiveness in infant‐centered family settings is hindered by domain mismatch. Adult speech data differs significantly from the complex, overlapping, and often less structured audio environments found in infant‐family home recordings. To mitigate this issue, W2V2‐LittleBeats (W2V2‐LB) was introduced as a domain‐adaptive variant of W2V2, specifically designed for infant and family audio analysis (Li et al. 2023). Unlike the original W2V2, which was pretrained on adult speech, W2V2‐LB was pretrained on 4300 h of infant‐centered home recordings. This adaptation allows the model to capture unique acoustic properties of home environments and vocalization patterns of parent–infant communication. This work is part of a broader shift toward domain‐adaptive self‐supervised models for naturalistic child‐family home audio, with recent approaches extending this strategy using larger multilingual corpora (e.g., BabyHuBERT, Charlot et al. 2025) or alternative encoder architectures (e.g., Band‐Split SSAMBA, Fan et al. 2025).

To situate the contribution of W2V2‐LB within the broader landscape of tools used in developmental research, it is important to contrast it with the LENA system, the most widely used platform for analyzing daylong home audio. LENA has played a critical role in scaling studies of early language environments; however, it presents several limitations for studying infant vocalizations in naturalistic settings. First, LENA was designed primarily to assess language input and development, providing broad speaker and vocalization categories (e.g., key child, adult speakers, child vocalizations, conversational turns), with a focus on speech‐like sounds, and its utility for assessing core dimensions of socioemotional development (e.g., caregiver responsiveness to distress cues) is limited. Although LENA provides cry labels, accuracy tends to be low, especially for infants under 6 months. For instance, in daylong home recordings, LENA's cry estimates were moderately correlated with human annotations over 5‐min segments (*r* = 0.58), underestimating cry duration by an average of 10 s (Micheletti et al. 2023). As such, researchers using the LENA platform typically rely on complementary human coding when studying infant crying. Second, LENA's speaker classification performance is modest: a systematic review reported mean recall and precision rates of 59% and 68%, respectively, and found that LENA detects only about half of the infant vocalizations identified by human coders (Cristia et al. 2020). Compared with recent DL approaches, LENA's algorithms rely on older statistical models for speech classification and diarization (Xu et al. 2009). This limits their ability to capture the complexity and variability of naturalistic infant‐caregiver interactions. In contrast, DL models have long outperformed statistical models in speech processing (Mehrish et al. 2023), and recent SFMs have achieved state‐of‐the‐art performance on several speech processing tasks with a limited amount of labeled data (Yang et al. 2021). Finally, LENA's closed‐source design restricts transparency, limits methodological flexibility, and prevents use with audio collected from nonproprietary recording devices.

Together, these limitations highlight a gap between the growing availability of large‐scale naturalistic infant–caregiver audio data and the analytic tools needed to study interaction processes that require precise speaker diarization, vocalization type classification, and temporal sequencing. By leveraging an open‐source, self‐supervised speech foundation model pretrained on infant‐centered home recordings, W2V2‐LittleBeats (W2V2‐LB) aims to provide a scalable, domain‐appropriate alternative to existing tools for analyzing complex infant‐family home audio.

### 2.1 Method

#### 2.1.1 Participants

A total of 61 infants aged 1.10–17.54 months (*M* = 9.43 months, *SD* = 3.52 months) and their parents participated in the study. Families were recruited through pediatric clinics and community organizations. With respect to demographic characteristics, 52% of infants were female, and 82% of mothers identified as White, 8% as Asian, 6% as Hispanic, 2% as Black, and 2% as another racial or ethnic background. English was the primary language spoken in 86% of households, while 14% of families spoke additional languages. Regarding maternal education, 4% of mothers had a high school or two‐year college degree, 32% held a bachelor's degree, and 64% had an advanced degree. The average annual family income fell within the $91,000–$100,000 range, although we note these data were missing for 11 families.

#### 2.1.2 Home Audio Recordings

We used LittleBeats, an infant‐wearable platform designed to capture synchronized audio, ECG, and IMU data, to collect home audio recordings. Parents were instructed on how to use LittleBeats and had access to online as well as written instructions as needed. LittleBeats is secured in the chest pocket of a specially designed infant shirt or onesie, and infants wore LittleBeats for 2‐3 nonconsecutive days (8+ h/day) over the course of 2 weeks. Prior reports have established the validity of the LittleBeats sensors against gold‐standard equipment (Islam et al. 2024) and have documented parents’ experiences related to ease of use, safety, and privacy protections when using LittleBeats in the home (McElwain et al. 2024).

#### 2.1.3 ML Approach

##### 2.1.3.1 Model Architecture

**FIGURE 1:**
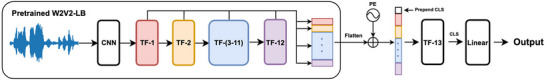
Study 1: Overall model architecture Note. CNN = convolutional neural network; TF = transformer encoder layer; PE = positional encoding; CLS = classify token.

For the current report, automatic speaker diarization and vocalization classification were performed using a system based on the pretrained W2V2‐LB model (Li et al. 2023, 2024). The overall model architecture is illustrated in Figure 1. W2V2‐LB is built on attention‐based context integration layers (Vaswani et al. 2017),  which are designed to learn speech representations from large‐scale unlabeled audio. In our system, we use W2V2‐LB representations and add an additional Transformer layer to model contextual information across time steps and layers before predicting diarization and vocalization classification labels.

We denote the output from all layers of W2V2‐LB as a speech embedding E with shape (L, T, F), where L is the number of layers, T is the number of temporal steps, and F is the feature dimension (see Figure 1). To aggregate information across layers, we first flatten the embedding along the layer dimension, resulting in a 2D representation of shape (L × T, F). A classification token (CLS) is then prepended, and positional embeddings (PE) are added to the sequence before it is passed through a Transformer encoder layer for final contextualization. The contextualized embedding of the CLS token is subsequently fed into a classifier layer to predict the target class.

For model training and evaluation, we partitioned the dataset into training, validation, and test sets, with data from each family represented in only one of these three sets. To give the model more context for understanding the audio and, thereby, improve performance, the recordings were segmented into overlapping 2‐s samples, using the surrounding context (0.95 s before and after) to predict the vocalization class in the central 0.1‐s interval. We finetuned W2V2‐LB for five epochs using cross‐entropy loss under the same setting as described in (Li et al. 2023), and the checkpoint yielding the highest Cohen's kappa score on the validation set was selected for final evaluation. The model's performance on the test set was assessed using accuracy, macro‐F1 score, Cohen's kappa, and class‐wise F1 scores. To facilitate reproducibility and reuse, the code and pretrained checkpoints for our wav2vec2‐based classification model are openly available at https://github.com/xulinfan/w2v_LB_Long_Transformer.

##### 2.1.3.2 Manual Annotation Used for Model Finetuning and Evaluation

The manual annotations used to finetune and evaluate the W2V2‐LB model were carried out by trained research assistants (RAs). RAs used Praat 6.4.05 (Boersma and Weenink 2024) to manually annotate audio recordings from the LittleBeats device. Each daylong recording was segmented into intervals (e.g., 30 s, 2 min, or 10 min), and we prioritized segments with the highest rates of infant and mother vocalizations for annotation, as estimated using the W2V2‐LB model. Trained RAs independently labeled each segment for speaker and vocalization types using onset‐offset coding, with separate tiers assigned for the focal infant (CHN), adult female (FAN), adult male (MAN), and older sibling/s (CXN). In cases of overlapping vocalizations, RAs annotated each speaker's vocalization on its own tier, and overlapping intervals were excluded from downstream analyses. For this report, we used the following five labels for training, validation, and testing: infant nondistress vocalizations (e.g., babbling, cooing), infant distress vocalizations (e.g., crying, fussing), adult female vocalizations (i.e., the mother), adult male vocalizations (i.e., the father), and silence/other (i.e., any noise that is not captured by the first four categories, including vocalizations from siblings).

To ensure annotation quality, 43% of the recording intervals were independently annotated by two RAs. Reliability of the manual annotation was high (accuracy = 0.89, macro‐F1 = 0.87, Cohen's kappa = 0.83). Additionally, individual class‐wise F1 scores indicate good to excellent reliability: infant nondistress vocalizations (0.80), infant distress vocalizations (0.91), adult female vocalizations (0.89), adult male vocalizations (0.83), and other (0.91).

### 2.2 Results

Our labeled dataset totals approximately 14 h of recordings. Table 1 shows the breakdown of the training, validation, and testing data sets with respect to the number of families included in each split, as well as the total number of minutes for all labeled data in each split and by category. Overall performance of the model on the test set was high (accuracy = 0.89, macro‐F1 = 0.78, Cohen's kappa = 0.76), and the full distribution of classification outcomes (confusion matrix) is presented in Table 2. Additionally, as shown in Table 3, individual class‐wise precision, recall, and F1 scores indicate good to excellent performance.

**TABLE 1:** Study 1: Training, validation, and testing splits in terms of sample sizes and active vocalization durations (in minutes).

				Adult vocalizations		Infant vocalizations	
Split	*N*	Total	Silence/other	Female	Male	Nondistress	Distress
Training	51	625.4	421.2	94.2	32.9	48.9	28.2
Validation	5	106.0	75.9	11.9	4.3	6.3	7.6
Testing	5	134.3	93.8	13.5	7.7	7.0	12.3

**TABLE 2:** Study 1: Confusion matrix for model performance.

True∖Pred	1	2	3	4	5
1. Silence/other	53699	1239	421	591	320
2. Adult–female	1700	5786	462	99	33
3. Adult–male	877	946	2739	17	22
4. Infant–nondistress	679	113	24	2855	559
5. Infant–distress	505	54	0	495	6317

*Note*: Rows represent ground truth labels and columns represent model predictions.

**TABLE 3:** Study 1: Model performance on the test set.

Class (vocalization type)	Precision	Recall	F1
Silence/other	0.94	0.95	0.94
Adult–female	0.71	0.72	0.71
Adult–male	0.75	0.60	0.66
Infant−nondistress	0.70	0.68	0.69
Infant−distress	0.87	0.86	0.86
**Macro‐average**	**0.79**	**0.76**	**0.78**

### 2.3 Discussion

Our proposed audio tagging method demonstrates the effectiveness of applying state‐of‐the‐art self‐supervised learning to infant‐centered audio analysis, specifically in the tasks of speaker diarization and infant vocalization classification. A major challenge in this domain is the mismatch between our real‐world recordings and the clean, controlled data used to train most existing speech models. Naturalistic recordings often include overlapping speech, background noise, and inconsistent recording conditions such as varying microphone placement, room acoustics, and device movement, all of which degrade the performance of the automated system. Leveraging large‐scale pretraining on unlabeled audio, the W2V2‐LB model learns noise‐robust, context‐aware representations that can be fine‐tuned efficiently on limited annotated data. This approach addresses challenges faced by fully supervised models in low‐resource settings (Cristia et al. 2021; Schuller et al. 2017; Xie et al. 2021), in which there is limited labeled data of parent‐infant vocalizations recorded at home for model training. Evaluation results indicate strong performance in identifying vocalizations from female caregivers and the focal infant, showing the model's ability to generalize well to the most common speaker classes.

Compared with widely used tools such as LENA, our open‐source W2V2‐LB model offers greater transparency, flexibility, and temporal precision for studying caregiver–infant vocal interactions. LENA operationalizes conversational turns using a default 5‐s window between adult and child vocalizations and imposes minimum vocalization duration thresholds (e.g., 600 ms for child vocalizations; Xu et al. 2009), with outputs typically aggregated at relatively coarse temporal scales (Richards et al. 2010). In contrast, W2V2‐LB supports high‐resolution, event‐based analyses at 0.1‐s granularity, allowing researchers to flexibly define lag windows and align brief vocal events with greater temporal accuracy. Moreover, it achieves comparable, and in some cases superior, accuracy in identifying female caregiver and infant vocalizations, and uniquely provides reliable labels for infant nondistress and distress vocalizations, a critical distinction not currently supported by the LENA system. Together, these features make W2V2‐LB particularly well suited for developmental research requiring fine‐grained, scalable analysis of naturalistic audio.

At the same time, it is important to clarify the scope of direct system comparisons. A direct comparison to the VTC system (Lavechin et al. 2020), for instance, was not feasible because of differences in label definitions and data sources. Our W2V2‐LB model presented here uses a more fine‐grained infant vocal category set (i.e., distress and nondistress vocalizations). In addition, our “OTH” category aggregates silence, nonspeech, and sibling speech, whereas VTC includes an “other child” speaker category. Future benchmarking efforts would benefit from harmonized annotation schemes and domain‐adaptation strategies to enable systematic cross‐model evaluation.

## 3 Study 2

Mothers’ vocal contingent responsiveness and mother–infant vocal turn‐taking (involving reciprocal contingent responses) have been posited to serve as an “engine of development” in which infants develop foundational communicative skills (Levinson 2016), a sense of agency (Gergely and Watson 1999), and core cognitive capacities (e.g., information processing, memory; Jaffe et al. 2001). Compared with mothers’ nonverbal contingent responses involving facial affect, gaze, or touch, mothers’ vocal contingent responses are theorized to present infants with salient demarcations between their own vocal behavior (nondistress vocalizations, in particular) and the mother's vocalizations. Such temporal alignment provides infants with important information that their vocalizations have predictable consequences.

To date, most evidence linking maternal vocal contingent responsiveness to infant outcomes is derived from brief laboratory or home‐visit observations, leaving open questions about how these interactional processes unfold across infants’ everyday environments and how they might be best quantified. Building on Study 1, we apply the validated W2V2‐LB algorithm to daylong (e.g., 8+ h) home recordings and quantify maternal responsiveness to infant vocalizations, which we refer to here as an event‐based metric. Although advances in (a) ML approaches permit automated diarization and labeling of audio and (b) infant wearables permit collection of extended daylong recordings in the home, these unprecedented opportunities to assess infants’ real‐world experiences of mothers’ vocal contingent responsiveness also come with challenges. In Study 2, we aim to shed light on three such challenges.

A first challenge in estimating maternal contingent responsiveness from daylong recordings is whether automated vocalization labels can be used to generate valid measures of interaction processes. Despite the growing use of automated speech‐processing systems (e.g., LENA and related diarization and classification pipelines), prior work has largely examined agreement with human annotations for vocalization labels and aggregate counts, rather than for explicitly defined interaction processes between infants and caregivers. Notably, when agreement metrics for interaction processes are assessed, they tend to be substantially lower than those for label‐based measures. For example, large‐scale LENA studies (e.g., Cristia et al. 2020) reported relatively low correspondence between automated and human annotations for conversational turn counts (*r* = 0.36), in contrast to higher agreement for adult word counts and child vocalizations (*r* = 0.77–0.79). Similarly, Ferjan Ramírez et al. (2021) reported that LENA systematically overestimated conversational turns compared with manual coding, with relative differences ranging from 72% to 745%. That is, in some cases, the LENA system was estimating approximately 8 conversational turns for every one turn identified by a human coder. Together, these findings raise a critical issue for studies using daylong recordings regarding whether ML‐generated labels can be used to provide valid estimates of interaction processes.

A second challenge concerns how behavior is sampled from the continuous audio stream. Because manual annotation of extended recordings is labor intensive, prior studies have adopted one of two broad approaches. One approach is to sample brief, high‐volubility segments, often identified using automated tools and followed by manual coding of infant and caregiver vocalizations (e.g., Lee and Ha 2023; Pretzer et al. 2019). A second approach leverages automated speech‐processing tools to analyze all available data from daylong recordings, thereby maximizing temporal coverage (e.g., Fields‐Olivieri and Cole 2019; Warlaumont et al. 2014). These sampling decisions have particularly salient consequences for estimates of more infrequent behaviors. For example, in a study that applied a DL model for automated cry detection among 27 infants, Micheletti et al. (2023) reported that estimates of infant crying were particularly sensitive to sampling coverage, with 5‐min sampling periods yielding more variable and less representative estimates than those aggregated over 1‐h and 24‐h periods. Likewise, in a second study of 11 preschool‐aged children, Micheletti et al. (2020) demonstrated that higher sampling coverage (12.5% vs. 0.5% of recordings) and systematic (vs. random) sampling reduced temporal clustering bias and produced behavior‐frequency estimates that more closely approximated estimates based on coding the entire daylong recording. These findings demonstrate the importance of evaluating how different approaches to sampling behavior may also shape estimates of caregiver–infant interaction processes.

A third challenge lies in the selection of a lag window used to determine whether a maternal vocalization following an infant cue is defined as contingent. Evidence from laboratory paradigms indicates that maternal vocal responses occur rapidly, often within 800 ms, which is consistent with infants’ biological capacity to detect contingencies (Keller et al. 1999). However, definitions of contingency in home‐based studies vary widely, with lag windows ranging from brief intervals of 1 s (e.g., Warlaumont et al. 2014), 2 s (e.g., Bornstein et al. 2015; Lee and Ha 2023), and 3 s (e.g., Bornstein and Manian 2013) to longer windows of up to 5 s (e.g., Pretzer et al. 2019; van Egeren et al. 2001). This variation poses a challenge because the chosen window directly affects estimates of contingent responsiveness (i.e., response rates will likely increase as the length of the lag window increases). Further, short lag windows risk missing meaningful but delayed caregiver responses, whereas longer windows increase the likelihood of capturing caregiver speech that is temporally proximal but not contingent to the infant's vocalization. Shedding light on this tradeoff, in a 25‐min home observation with researchers present, van Egeren et al. (2001) showed that the likelihood of maternal vocal response to infant vocalization is front‐loaded in time, with cumulative Yule's Q values (a base‐rate‐adjusted estimate of contingency) increasing sharply from 1 s to 3 s and plateauing thereafter. Yet, van Egeren et al. (2001) relied on a relatively short and semi‐structured observational session, leaving open the question of how differing lag windows affect estimates of maternal contingency in daylong home recordings.

Taken together, the three challenges outlined above provide important context for the assessment of maternal vocal contingent responses from home audio recordings. In Study 2, we aim to shed light on each of these challenges, in turn, by (a) evaluating agreement between maternal contingent response rates computed from W2V2‐LB vocalization labels versus human annotations, (b) comparing the distributions of response rates derived from different strategies for sampling behaviors (i.e., the 20 most voluble 5‐min segments versus all available mother–infant segments), and (c) examining how contingency estimates (both response rate and base‐rate‐adjusted estimates) vary as a function of behavioral sampling strategy and lag window (i.e., 1 s, 2 s, 3 s, 4 s, 5 s). Because infant nondistress and distress vocalizations differ in terms of frequency (e.g., Barr 1990) and function (e.g., Bell and Ainsworth 1972; Donnellan et al. 2020) and because mothers’ contingent responsiveness may differ as a function of vocalization type (e.g., Pretzer et al. 2019), we examined infant nondistress and distress vocalizations separately in addressing the above aims.

### 3.1 Method

#### 3.1.1 Participants

A total of 56 infants (48% female; 66% firstborn) aged 1–10 months (*M* = 6.51 months, *SD* = 1.98 months) and their parents participated. Families were recruited through pediatric clinics, local organizations serving families with young children, and online forums. Sixty‐seven percent of mothers identified as White, 13% as Asian, 11% as Hispanic, 6% as Black, and 3% as other racial or ethnic backgrounds. English was the sole language spoken in the home for 68% of families, whereas other languages in addition to English were spoken in 32% of homes. Six percent of mothers had a high school or 2‐year college degree, 29% had a bachelor's degree, and 64% had an advanced degree. Thirty‐two families were included in both Study 1 and Study 2.

#### 3.1.2 Data Collection and Processing Procedures

Paralleling Study 1 procedures, infants wore LittleBeats in the home across 2‐3 nonconsecutive days within a two‐week period. The ML algorithm described and validated in Study 1 was applied to the daylong recordings. To facilitate the selection of audio segments for subsequent analyses, daylong recordings were divided into contiguous 5‐min segments. To minimize false positives (e.g., detecting contingency when the mother was speaking to the father rather than the infant), we selected 5‐min segments that included only mother–infant vocalizations (i.e., mother vocalizations ≥ 2 s, infant nondistress + distress vocalizations ≥ 2 s, and father vocalizations < 2 s).

We applied lag windows of 1 s, 2 s, 3 s, 4 s, and 5 s to extract maternal response rates. A contingent response was defined as any maternal vocalization that began within the specified lag following the offset of an infant vocalization (see Northrup and Iverson 2020), and we used a one‐to‐one mapping rule to ensure unique pairing between infant and maternal vocalizations (see Farran et al. 2019; Pretzer et al. 2019). Specifically, when multiple infant vocalizations occurred before the same mother vocalization, the maternal vocalization was paired with the most recent preceding infant vocalization. Likewise, when several maternal vocalizations occurred after the same infant vocalization, the maternal vocalization closest in time to the infant vocalization and within the specified lag window was coded as contingent. Infant vocalizations shorter than 0.5 s were excluded from this analysis, as such brief events are presumed less likely to represent meaningful communicative behaviors. For each 5‐min segment, the response rate was calculated separately for infant nondistress and distress vocalizations and was defined as the proportion of vocalizations that received a maternal response within the lag window (i.e., number of infant vocalizations followed by a maternal vocalization divided by the total number of infant vocalizations of that type in the 5‐min segment). If no infant vocalizations of a given type (i.e., nondistress, distress) occurred, the response rate for that type was coded as missing.

#### 3.1.3 Data Analytic Plan

To address our first aim, we compared maternal contingent response rates computed from ML‐generated versus human‐coded vocalization labels. Among the 32 Study 2 families who were also included in Study 1, human coders annotated 47 30‐s segments and 37 10‐min segments from the daylong audio recordings. We included only the 10‐min segments (≈6.17 h from 12 families) for this analysis to more closely parallel analyses conducted for subsequent Study 2 aims that use 5‐min segments. For each family, we aggregated all available 10‐min segments to compute maternal response‐rate scores separately for ML‐labeled and human‐coded data. Agreement between the two approaches was evaluated using Bland–Altman analyses including (a) mean difference (i.e., average difference between response rates computed from ML‐derived and human‐coded vocalization labels) and its corresponding confidence interval (CI), which indexes systematic bias and indicates whether one method tends to overestimate or underestimate the other across observations, (b) relative (log‐scale) difference derived from the geometric mean ratio (ML/Human), which assesses relative agreement under potential heteroscedasticity, and (c) limits of agreement (mean difference ± 1.96 *SD* of the differences), which quantifies the range within which most differences between methods are expected to fall. Given our sample size of 12, Bland–Altman analysis served as the primary agreement metric because it is robust for small samples (Bland and Altman 1986). For descriptive purposes, Pearson correlations were also computed, and scatterplots were generated to visualize these associations, consistent with recommendations for small sample sizes (Weissgerber et al. 2015).

To address our second and third aims, we used ML‐generated labels for our full Study 2 sample (*N* = 56). First, we compared maternal response rates derived from (a) all available 5‐min segments that met our criteria for computing maternal contingent response rate (*n* = 2556) and (b) a subset of 20 five‐min segments (per family) with the highest volubility based on the total duration of maternal and infant vocalizations (*n* = 1046) for each lag window (i.e., 1 s, 2 s, 3 s, 4 s, 5 s). We performed Kolmogorov–Smirnov (K‐S) tests on the distributions of the maternal response rate across these two datasets, yielding 10 comparisons (5 lag windows × 2 infant vocalization types). A significant *p*‐value indicates that the distributions differ, suggesting that the subset (i.e., segments with highest volubility) is not representative of the dataset with all available 5‐min segments. To aid interpretation, we present probability density plots that contrast response‐rate distributions from the two datasets. We note that 11 families had fewer than 20 5‐min segments containing only mother and infant vocalizations: five families had 15–19 segments, four families had 10–14 segments, and two families had 6‐7 segments. Therefore, we conducted sensitivity analyses with these families excluded.

Next, we compared estimates of maternal response rates as a function of behavioral sampling strategy and lag window. Because daylong home recordings include large fluctuations in infant and maternal vocalizations across contexts, reliance on raw response rates alone may confound response rate estimates with overall vocal density. To address this issue, we computed Yule's Q (a base‐rate‐adjusted transformation of the odds ratio derived from 2 × 2 contingency tables) to provide a rigorous estimate of maternal contingent responsiveness. This approach extends prior work on maternal responsiveness in a 25‐min home‐based observational session (e.g., van Egeren et al. 2001), where responsiveness was evaluated using lag‐sequential methods, Yule's Q to control for base rates, and cumulative analyses across successive seconds to determine when contingency plateaued. Yule's Q can range from −1 to +1 and indexes whether maternal vocalizations are more likely (positive values) or less likely (negative values) to occur within the specified lag window following infant vocalization onset than at other times, while accounting for overall infant and maternal vocalization base rates. For each infant vocalization type and sampling strategy, we discretized each segment into 0.1‐s time bins and classified each bin according to (a) whether it fell within the specified lag window (1–5 s) following an infant vocalization onset (cue present versus absent) and (b) whether a maternal vocal onset occurred within that bin (response present versus absent). Separate 2 × 2 contingency tables were constructed for each lag window. To further assess lag‐window patterns of base‐rate‐adjusted estimates (Yule's Q), we fit four multilevel polynomial mixed models (2 behavioral sampling strategies × 2 vocalization types) in which lag window (1–5 s) was treated as a repeated measure within segment, with segments nested within infants. Linear and quadratic lag terms were included to test whether contingency showed linear increases and/or curvature across windows.

### 3.2 Results

#### 3.2.1 Maternal Contingent Response Rates Using Machine‐Learning Versus Human‐Coded Labels

**FIGURE 3:**
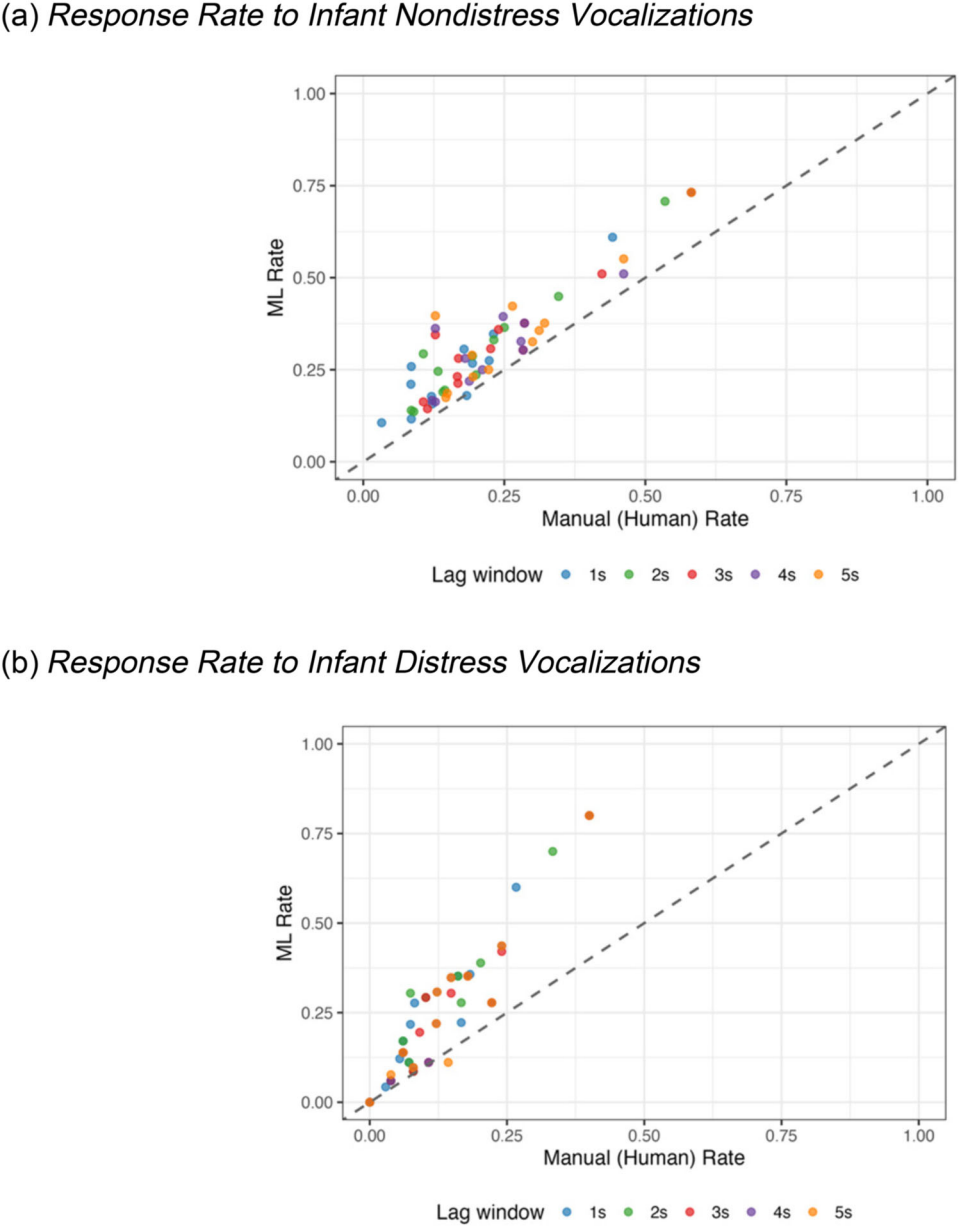
Study 2: Scatterplots of ML–derived and human‐coded maternal contingent response rates to infant nondistress and distress vocalizations (*N* = 12) across 1‐, 2‐, 3‐, 4‐ and 5‐s lag windows. Note. Points represent family‐level estimates. The dashed diagonal line indicates perfect agreement.

First, using data from a subsample of 12 families, we compared contingent response rates computed from ML‐generated vocalization labels and human‐coded labels under identical analytic settings (i.e., 1–5 s lag windows, one‐to‐one mapping, exclusion of infant vocalizations < 0.5 s). Bland–Altman plots (Figure 2) show consistently positive mean differences across lag windows for both vocalization types, ranging from 0.082 to 0.093 for nondistress and 0.111 to 0.128 for distress vocalizations. Corresponding 95% CIs were above zero (see Table 4). Relative (log‐scale) difference ranged from 34% to 64% for nondistress vocalizations and from 65% to 95% for distress vocalizations, indicating that response rates computed from W2V2‐LB–derived labels were, on average, one‐third to two‐thirds higher for nondistress vocalizations and about two‐thirds to nearly double for distress vocalizations. Complementing the Bland–Altman analyses, correlations between response rate computed by ML‐ and human‐coded labels were consistently high (*r*s ≥ 0.89, see Table 4) and scatter plots showed strong linear correspondence with points closely clustering around the diagonal line of agreement (see Figure 3). In sum, despite a consistent upward bias in absolute levels, ML‐derived labels yield response rates that show strong rank‐order correspondence to those obtained from human annotation.

FIGURE 2Study 2: Bland–Altman plot of ML‐derived and human‐coded maternal contingent response rates to infant nondistress and distress vocalizations (*N* = 12) across 1‐, 2‐, 3‐, 4‐, and 5‐s lag windows.Note. The blue line denotes the mean difference; red dashed lines indicate ± 1.96 *SD*. Points represent family‐level estimates.

**Figure:**
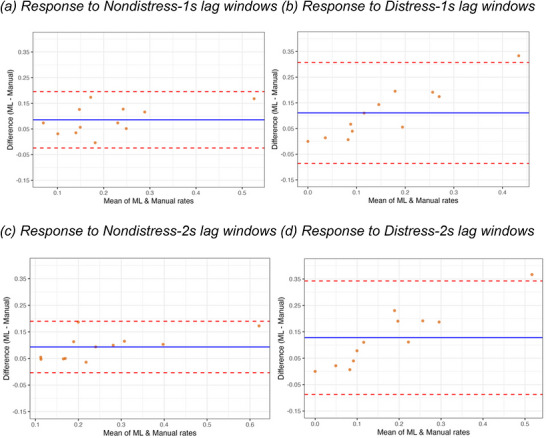

**Figure:**
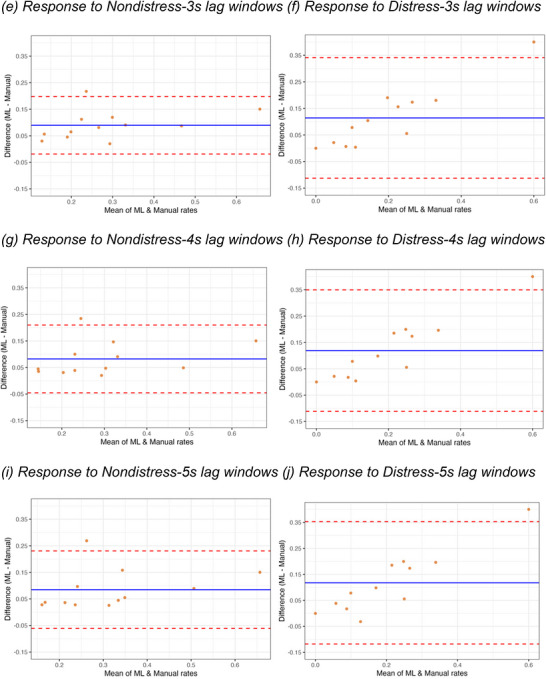

**TABLE 4:** Study 2: Agreement between ML‐derived and human‐coded maternal response rates by infant vocalization type and lag window.

Vocalization type	Lag	Pearson *r*	Mean difference (*SD*)	95% CIs of mean difference	Relative difference	Lower LoA	Upper LoA
Nondistress vocalizations	1s	0.919	0.086 (0.056)	(0.050–0.121)	64.014%	−0.024	0.196
	2s	0.962	0.093 (0.049)	(0.062–0.125)	51.361%	−0.004	0.190
	3s	0.943	0.090 (0.055)	(0.054–0.125)	41.945%	−0.019	0.198
	4s	0.912	0.082 (0.065)	(0.041–0.124)	35.600%	−0.045	0.210
	5s	0.885	0.085 (0.074)	(0.038–0.132)	33.589%	−0.061	0.231
Distress vocalizations	1s	0.926	0.111 (0.100)	(0.047–0.175)	88.427%	−0.086	0.308
	2s	0.943	0.128 (0.110)	(0.058–0.197)	94.547%	−0.087	0.343
	3s	0.953	0.114 (0.116)	(0.041–0.188)	65.944%	−0.113	0.341
	4s	0.956	0.119 (0.118)	(0.044–0.194)	65.771%	−0.111	0.350
	5s	0.939	0.118 (0.120)	(0.041–0.194)	65.269%	−0.118	0.353

*Note*: Mean difference = response rate computed from ML‐labeled data − response rate computed from human‐coded data; 95% CIs of the mean difference = confidence interval for the mean difference, reflecting uncertainty around mean difference; Relative difference = relative difference derived from log‐scale Bland–Altman analyses, calculated as the percent difference corresponding to the geometric mean ratio (ML/Human); LoA = limits of agreement; lower LoA and upper LoA indicate the lower and upper bounds of the 95% limits of agreement, respectively.

#### 3.2.2 Distributions of Maternal Contingent Response Rates as a Function of Behavioral Sampling Strategy

Next, using data from our full Study 2 sample (*N* = 56), we compared the distribution of contingent response rates computed from all available 5‐min segments and a subset of 20 most voluble five‐min segments. Across all lag windows, K‐S tests showed significant differences in distributions: maternal contingent response rates to infant nondistress, *D*s (1040, 2524) = 0.09–0.11, *p*s < 0.001, and infant distress, *D*s (782, 1846) = 0.10–0.12, *p*s < 0.001. As shown in the probability density plots in Figure 4, response‐rate distributions from the 20 most voluble segments (compared with all available segments) tended toward higher values, with relatively little density at low response rates. In contrast, all available segments showed density distributed across a wider range of response rates, including lower values. In sensitivity analyses excluding families with fewer than 20 segments, K‐S tests continued to indicate significant differences, *D*s (896, 2380) = 0.12–0.13 for nondistress vocalizations and *D*s (708, 1772) = 0.12–0.14 for distress vocalizations, all *p*s < 0.001. Together, these results indicate that sampling only highly voluble segments may overrepresent interactions characterized by greater maternal engagement, whereas using all available segments captures a wider spectrum of interaction contexts.

FIGURE 4Study 2: Comparison of probability density functions between all segments and the 20 most voluble segments across 1‐, 2‐, 3‐, 4‐, and 5‐s lag windows.Note. The probability density is shown in blue for all segments and in green for the subset of most voluble segments. Kolmogorov–Smirnov tests showed significant distribution differences between full‐day and top‐20 segments across all lag windows and both vocalization types (*D*s = 0.09–0.12, *p*s < 0.001).

**Figure:**
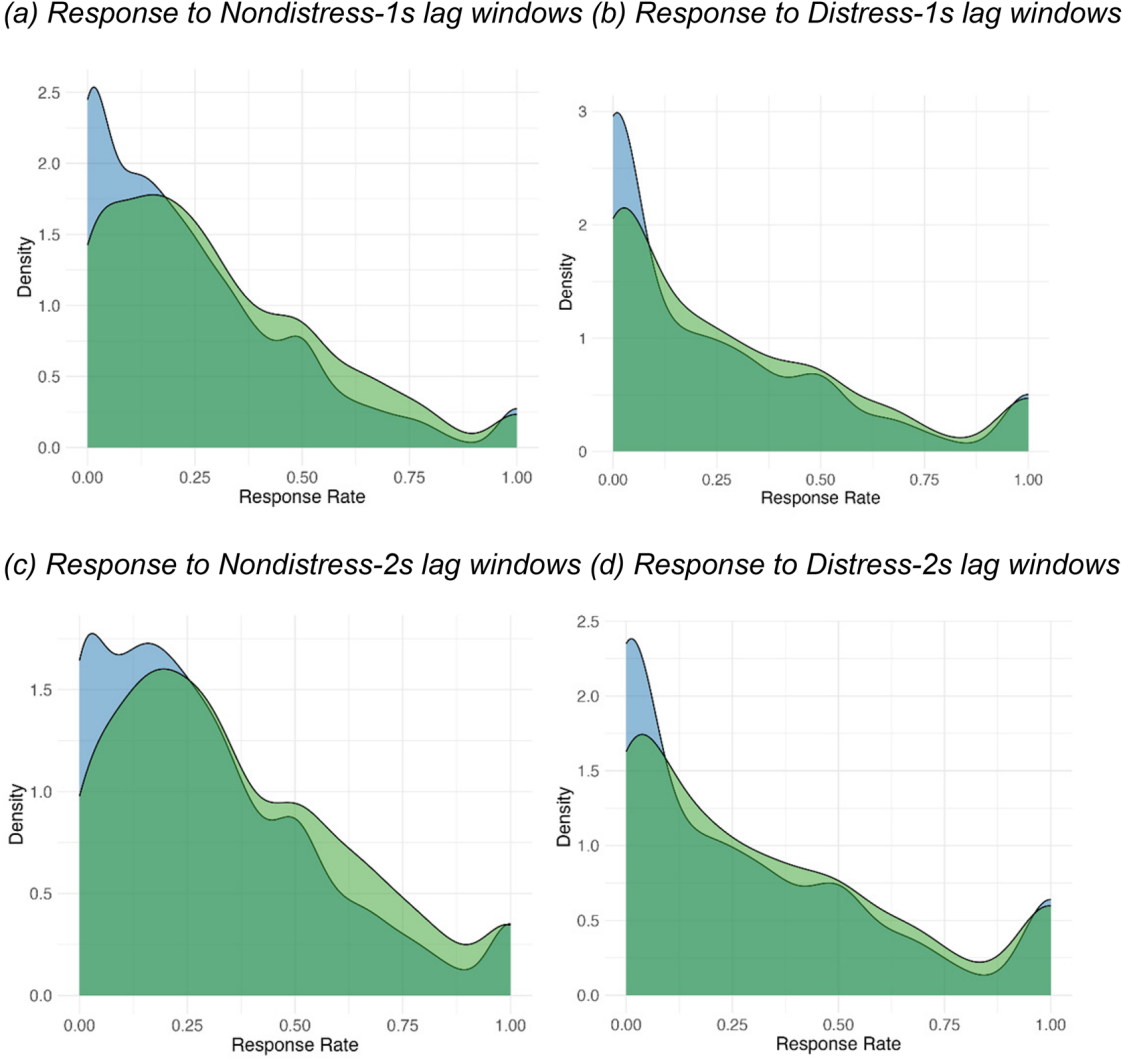

**Figure:**
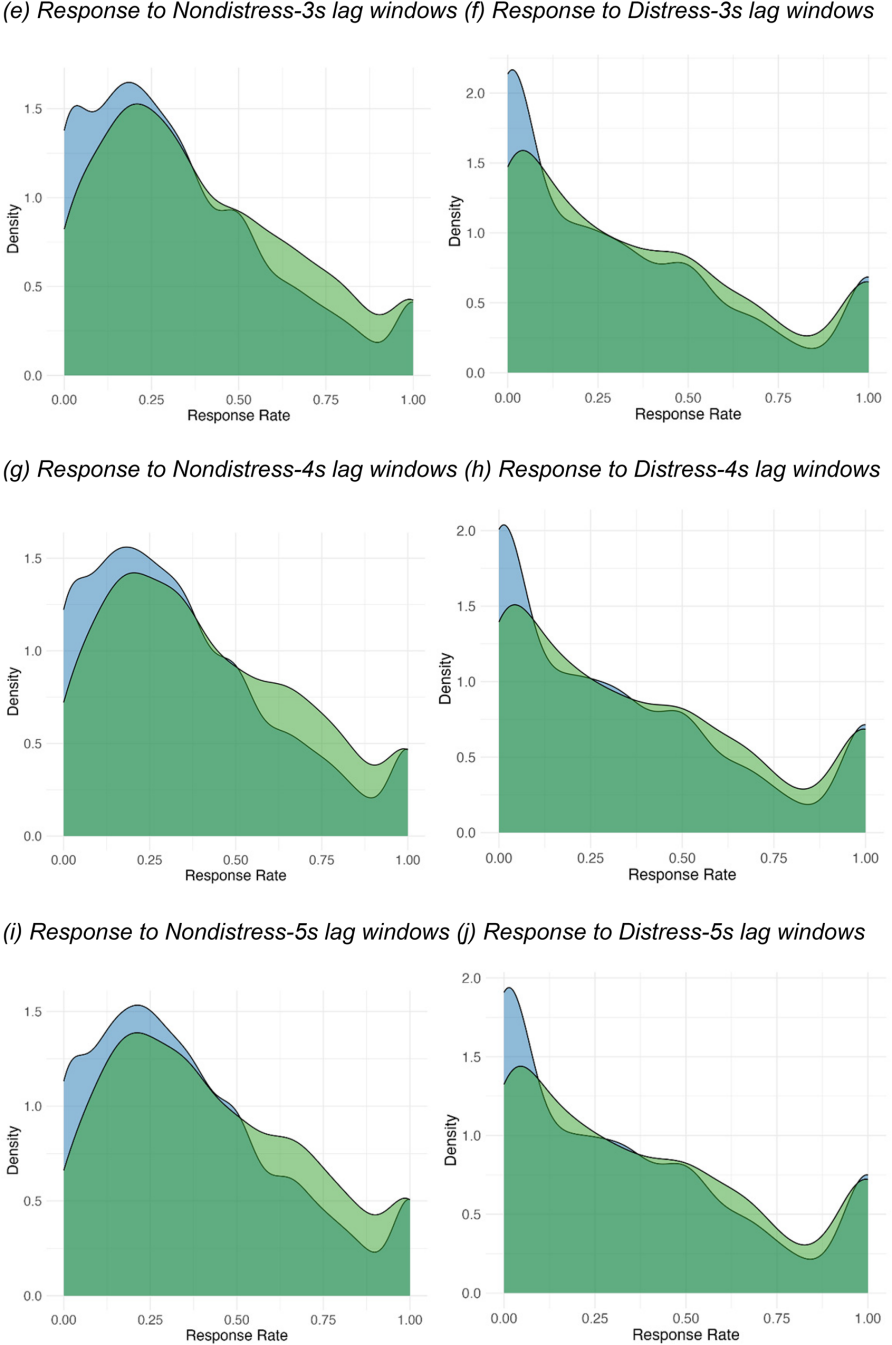

#### 3.2.3 Mean Levels and Base‐Rate‐Adjusted Estimates of Maternal Contingency as a Function of Behavioral Sampling Strategy and Lag Window

**FIGURE 5:**
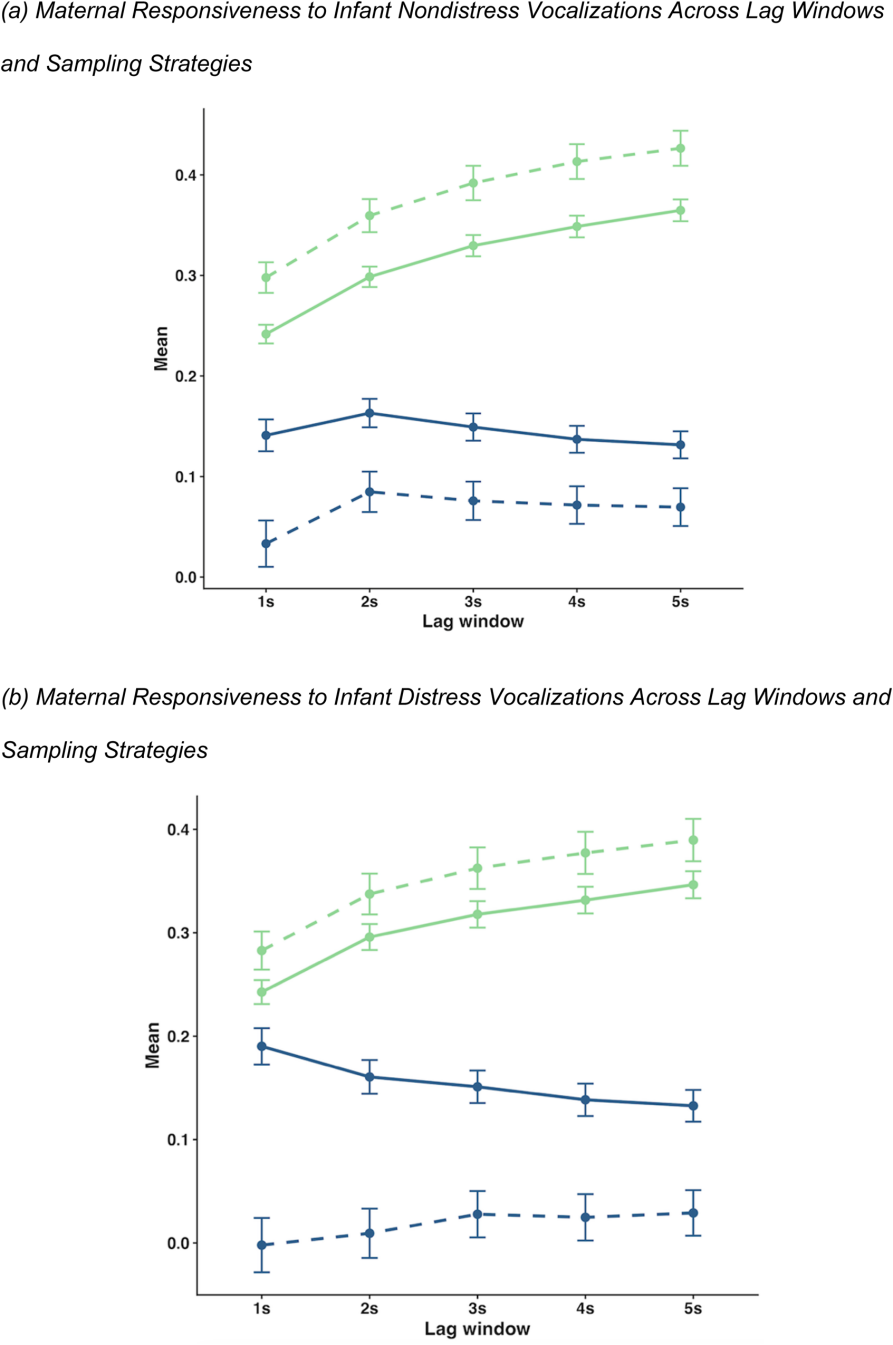
Study 2: Response rates and base‐rate‐adjusted estimates (Yule's Q) by lag window for infant nondistress versus distress vocalizations. Note. Blue lines represent base‐rate‐adjusted estimates (Yule's Q), and green lines represent mean maternal response rate. Solid lines indicate estimates based on all available segments, and dashed lines indicate estimates based on the 20 most voluble segments. Points represent segment‐level means, and error bars denote 95% confidence intervals.

Lastly, we examined how estimates of maternal contingency varied across behavioral sampling strategy and lag windows (1–5 s) for the full Study 2 sample. Segment‐level means and standard deviations for maternal response rates and base‐rate‐adjusted estimates are shown in Table 5, with corresponding patterns illustrated in Figure 5. Patterns in mean response rates differed by behavioral sampling strategy, independent of lag window and infant vocalization type, and were consistently higher in the 20 most voluble segments than in all available segments. In contrast, base‐rate‐adjusted estimates of contingency were generally higher for all available segments than for the 20 most voluble segments.

**TABLE 5:** Study 2: Response rates and base‐rate‐adjusted estimates (Yule's Q) by lag window for infant nondistress versus distress vocalizations.

		Nondistress vocalization		Distress vocalization	
Dataset	Lag	Response rate *M* (*SD*)	Yule's Q *M* (*SD*)	Response rate *M* (*SD*)	Yule's Q *M* (*SD*)
20 most voluble mother–infant segments	1s	0.30 (0.25)	0.03 (0.38)	0.28 (0.30)	−0.002 (0.43)
	2s	0.36 (0.27)	0.08 (0.33)	0.34 (0.33)	0.01 (0.39)
	3s	0.39 (0.28)	0.08 (0.32)	0.36 (0.33)	0.03 (0.37)
	4s	0.41 (0.29)	0.07 (0.31)	0.38 (0.34)	0.02 (0.37)
	5s	0.43 (0.29)	0.07 (0.31)	0.39 (0.34)	0.03 (0.36)
All available mother–infant segments	1s	0.24 (0.24)	0.14 (0.41)	0.24 (0.30)	0.19 (0.45)
	2s	0.30 (0.26)	0.16 (0.36)	0.30 (0.32)	0.16 (0.42)
	3s	0.33 (0.27)	0.15 (0.35)	0.32 (0.33)	0.15 (0.40)
	4s	0.35 (0.28)	0.14 (0.35)	0.33 (0.33)	0.14 (0.40)
	5s	0.36 (0.28)	0.13 (0.35)	0.35 (0.34)	0.13 (0.40)

*Note*: For the 20 most voluble segments, nondistress segments *n* = 1040 and distress segments *n* = 782. For all available segments, nondistress segments *n* = 2524 and distress segments *n* = 1846.

With regard to lag windows, results depended on infant vocalization type (see Table 5 and Figure 5). For nondistress vocalizations, the largest increase in mean maternal response rate occurred between 1 s and 2 s, and base‐rate‐adjusted estimates showed a similar early concentration of contingency, followed by plateauing or small decreases at longer lag windows. This pattern was observed for data from both behavioral sampling strategies. Polynomial mixed models for base‐rate‐adjusted estimates confirmed this pattern, yielding significant linear and quadratic lag effects: linear, *F*(1, 4158) = 13.74, *p* < 0.001, and quadratic, *F*(1, 4158) = 29.10, *p* < 0.001, terms for the 20 most voluble segments; linear, *F*(1, 10094) = 17.39, *p* < 0.001, and quadratic, *F*(1, 10094) = 17.61, *p* < 0.001, terms for all available segments.

For infant distress vocalizations, similar to the pattern for nondistress vocalizations, mean maternal response rate showed the largest increase between 1 s and 2 s for both all‐available and most‐voluble segments. However, base‐rate‐adjusted estimates of contingency showed divergent patterns depending on the behavioral sampling strategy. Specifically, base‐rate‐adjusted contingency increased gradually from near zero at 1 s to higher values at 3–5 s using the 20 most voluble segments. In contrast, Yule's Q was highest at 1 s and showed decreases at longer windows for all available segments. Corresponding polynomial models for base‐rate‐adjusted estimates indicated that for the 20 most‐voluble segments, lag effects were primarily linear, *F* (1, 3126) = 13.16, *p* < 0.001, with a nonsignificant quadratic term, *F* (1, 3126) = 1.99, *p* = 0.159. For all available segments, however, lag effects included a strong linear term, *F* (1, 7382) = 94.61, *p* < 0.001 and a smaller but significant quadratic term, *F* (1, 7382) = 7.10, *p* = 0.008.

### 3.3 Discussion

We highlight three key findings of Study 2. First, Bland–Altman analyses indicated a positive bias (ML > human), which is consistent with prior validation work on automated daylong‐audio systems used for interaction metrics. Notably, the magnitude of relative difference observed in our study (34%–95%) is substantially smaller than that reported by Ferjan Ramírez et al. (2021) for LENA‐derived conversational turn counts (72%–745% average overestimation). Nonetheless, this positive bias requires explanation. One possible contributor to the positive bias is that overlapping speech is handled differently in the automated and human coding systems. In the human annotations, caregiver vocalizations that begin before an infant vocalization has ended are treated as overlapping speech and do not count as contingent responses under our definition (i.e., responses must begin after infant offset and within the lag; also see Northrup and Iverson 2020). In contrast, W2V2‐LB outputs nonoverlapping speaker segments. When infant and caregiver speech overlap acoustically, the model may represent them as sequential rather than simultaneous events, making caregiver speech that began during the infant vocalization appear to start immediately after it ended, thereby inflating response rates computed by ML‐generated labels. Additional contributors include misclassification of maternal vocalizations directed to a sibling as infant‐directed responses, as well as greater temporal sensitivity of the model relative to human annotation. With respect to the latter concern, small onset/offset boundary differences (reflecting the model's finer temporal resolution, sometimes discussed in relation to over‐segmentation in automated systems) may shift events across lag thresholds, increasing the likelihood that ML‐detected vocalizations fall within the response window.

Despite this positive bias in absolute response rates, maternal responsiveness derived from W2V2‐LB labels showed strong rank‐order correspondence with human‐coded estimates (*r*s ≥ 0.89). Importantly, this agreement was observed under a conservative validation setup that retained acoustically complex segments, including those with overlapping vocalizations and adult male speech. The strong rank‐order correspondence observed under these challenging conditions further supports the validity of W2V2‐LB labels for later analyses, which applied duration‐based exclusions to more narrowly isolate maternal–infant interaction by removing segments with extended adult male speech.

Second, the distribution of maternal response rates varied systematically as a function of the strategy for sampling behavior. For both infant nondistress and distress vocalizations, response rates derived from the 20 most voluble segments were consistently higher than those based on all available segments. This pattern suggests that the distribution of response rates is conditional on how vocal behavior is sampled, as vocalizations are unevenly distributed across time and across families, and does not imply that either approach is inherently optimal. Rather, these sampling parameters are useful to consider in the context of the research goal. Sampling high‐volubility segments may be particularly informative when the goal is to characterize maternal responsiveness during periods of active dyadic exchange, when vocal events are frequent and contingency estimates are based on a larger number of cue–response observations within a comparable window (e.g., Endevelt‐Shapira et al. 2024; Lee and Ha 2023; Pretzer et al. 2019). In contrast, analyses based on all available segments may be preferable when the goal is to quantify infants’ overall exposure to vocal interactions in the home environment and the typical ebb and flow of vocal interactions across the day, including low‐ activity contexts (e.g., feeding, soothing, diapering, or transitional periods) that may nevertheless contribute to individual differences in infants’ socioemotional and language development (e.g., Ramírez‐Esparza et al. 2014; Warlaumont et al. 2014). Researchers should therefore consider how their behavioral sampling strategy aligns with their research questions and exercise caution when comparing response‐rate estimates across studies that adopt different sampling approaches.

Third, mean response rate and base‐rate‐adjusted estimates of contingency varied across behavioral sampling strategy and lag windows (1–5 s). Mean response rates are directly comparable to prior work but are sensitive to how often infants and mothers vocalize within a segment. In contrast, base‐rate‐adjusted estimates control for these marginal frequencies, making estimates more comparable across segments that differ in overall vocal activity, which is especially important in daylong home recordings where vocalization density fluctuates widely. Differences between sampling strategies reflected these properties. Selecting highly voluble segments concentrates periods of dense vocal activity from both infants and mothers, increasing the likelihood that maternal speech will fall within the response window due to event clustering rather than stronger temporal coupling (Bakeman and Gottman 1997). Response rates therefore increase as a function of vocalization density, whereas base‐rate‐adjusted estimates discount the elevated likelihood of chance overlap when both partners are vocalizing frequently. This pattern highlights the value of considering both metrics when interpreting contingent responsiveness in naturalistic data.

Regarding lag windows, across both sampling strategies and vocalization types, longer lag windows yielded higher overall response rates by capturing more delayed maternal responses, and gains showed diminishing returns beyond 2 s. Base‐rate‐adjusted estimates, however, revealed a more nuanced pattern that differed by vocalization type. For nondistress vocalizations, base‐rate‐adjusted estimates were strongest at the 2‐s window and showed diminishing gains beyond this point. This pattern is consistent with prior work showing that infant–adult vocal contingencies for speech‐like vocalizations are strongest within 1‐2 s windows (Pretzer et al. 2019) and with findings that maternal responsiveness peaks within the first few seconds (around 2‐3 s) and then plateaus at longer lag windows (van Egeren et al. 2001). For distress vocalizations, however, lag windows depended more on the sampling strategy. When all available segments were included, the base‐rate‐adjusted estimate was highest at the shortest lag window, consistent with the attachment‐relevant function of distress signals, which function to elicit rapid caregiver comfort and support (Bell and Ainsworth 1972). Within high‐volubility segments, however, contingency continued to increase up to 3 s, a pattern consistent with prior findings that reflexive vocalizations (e.g., cries) can remain predictive of adult responses at longer time windows than speech‐like vocalizations (Pretzer et al. 2019). These segments likely reflect periods of dense, ongoing interaction in which maternal responses to distress are embedded within a broader mother–infant exchange rather than occurring as discrete “repair” responses. In such contexts, slightly longer response windows may reflect the natural pacing of sustained interaction rather than changes in underlying contingency. Taken together, a lag window around 2 s appears to balance capturing early contingency and limiting inflation from extended windows for nondistress vocalizations, whereas the lag window used to capture contingent response to infant distress vocalizations may depend on interactional contexts.

## 4 General Discussion

Our study bridges ML and event‐based approaches by examining maternal contingent responsiveness to infant vocalizations in naturalistic home environments. Study 1 establishes that ML‐generated vocalization labels provide a valid foundation for examining maternal contingent responsiveness in daylong recordings. Building on this foundation, Study 2 demonstrates that maternal contingent response rates computed from ML‐generated vocalization labels preserve individual differences computed from human annotations and shows that analytic decisions—such as how vocal behavior is sampled and how the lag window is defined—systematically shape estimates of maternal contingent responsiveness.

Beyond demonstrating the feasibility of automated labeling, this work advances developmental science by showing how ML tools can be integrated with event‐based frameworks for studying caregiver–infant interaction. Rather than treating automated outputs as coarse substitutes for human coding, we illustrate that ML‐derived vocalization labels can support theoretically interpretable measures of contingency when paired with principled analytic definitions. This integration enables analysis at temporal and ecological scales not feasible through manual coding. Further, by showing that sampling strategy and lag‐window selection systematically alter both raw response rates and base‐rate‐adjusted estimates, we suggest that contingency estimates derived from daylong audio recordings are not purely properties of caregiver behavior, but also of the temporal and interactional distribution of vocal activity. In our data, periods of dense vocal exchange inflate raw response rates, whereas base‐rate‐adjusted metrics reveal different patterns, and lag‐window effects vary by vocalization type and sampling strategy. These findings indicate that analytic choices directly shape estimates of contingency strength, underscoring the need for transparent, theoretically informed decisions when using ML‐derived labels to quantify caregiver–infant interaction in naturalistic settings.

Despite these contributions, we note several limitations. First, W2V2‐LB collapses silence and other nontarget sounds, including sibling vocalizations, into a single class. As a result, some maternal vocalizations directed to siblings may be counted as infant‐directed responses, inflating response rates. Relatedly, the model lacks dedicated categories for other adult female speakers, so their vocalizations may be attributed to the mother, further inflating contingency estimates. Second, the homogeneous sample characterized by predominantly high‐SES, highly educated mothers, limits generalizability. Socioeconomic stressors may deplete caregivers’ cognitive and emotional resources for contingent responsiveness, whereas higher SES often acts as a protective factor, buffering stressors through access to parenting resources that facilitate timely responses (Bradley and Corwyn 2002). Future research should assess performance of automated vocalization detection and contingency estimation across more diverse samples, as variations in home environments (e.g., background noise, household composition) may affect decisions for quantifying maternal contingent responsiveness. Third, applying a fixed lag across all infant vocalizations may not fully reflect the variability in caregiver availability and responsiveness throughout the day. Parents may respond immediately when nearby but exhibit longer delays when multitasking or in another room. Adaptive lag models (e.g., dyad‐specific or context‐sensitive windows) could better reflect these natural interaction rhythms. Similar approaches have been used in ERP research, where analyses accommodate individual differences in neural response timing (e.g., Hu et al. 2009). Finally, our analyses, which treat infant vocalizations as triggers for caregiver responses, implicitly assume a unidirectional association from infant to mother. Yet, infant‐caregiver interactions are bidirectional, and caregiver behavior also shapes infant vocalization patterns (e.g., Pretzer et al. 2019; van Egeren et al. 2001). Future studies specifically designed to test bidirectional effects would complement the current findings.

In sum, ML approaches, when thoughtfully integrated with developmental constructs and theory, can advance our understanding of the fine‐grained temporal dynamics of early mother–infant vocal interactions. Our findings provide a scalable framework for quantifying maternal contingent responsiveness to infants’ vocalization from daylong home audio recordings. This integration of computational and developmental approaches represents a promising direction for investigating the complex social processes that shape early development, ultimately enhancing our understanding of infant development in real world environments.

## Author Contributions

**Kexin Hu**: conceptualization, methodology, formal analysis, data curation, writing – original draft, visualization. **Xulin Fan**: conceptualization, methodology, software, formal analysis, validation, writing − original draft. **Yannan Hu**: conceptualization, methodology, visualization, writing − review and editing. **Jialu Li**: conceptualization, methodology, software, writing − review and editing. **Bethany Lee**: project administration, data curation. **Mark Hasegawa‐Johnson**: resources, supervision, funding acquisition, writing ‐ review and editing. **Nancy L. McElwain**: conceptualization, resources, project administration, supervision, funding acquisition, writing − original draft.

## Conflicts of Interest

The authors declare no conflicts of interest.

## Data Availability

The raw home audio recordings analyzed in this study cannot be made publicly available due to institutional review board (IRB) restrictions and participant privacy protections. However, the machine learning model code, pretrained model checkpoints, and documentation necessary to reproduce the automated vocalization classification procedures are made publicly available via the project's GitHub repository. Information about accessing these materials is provided in the manuscript.
